# Emerging challenges for sustainable development and forest conservation in Sarawak, Borneo

**DOI:** 10.1371/journal.pone.0229614

**Published:** 2020-03-03

**Authors:** Mohammed Alamgir, Mason J. Campbell, Sean Sloan, Jayden Engert, Jettie Word, William F. Laurance

**Affiliations:** 1 Centre for Tropical Environmental and Sustainability Science, College of Science and Engineering, James Cook University, Cairns, Queensland, Australia; 2 The Borneo Project, Earth Island Institute, Berkeley, CA, United States of America; University of Georgia, UNITED STATES

## Abstract

The forests of Borneo—the third largest island on the planet—sustain some of the highest biodiversity and carbon storage in the world. The forests also provide vital ecosystem services and livelihood support for millions of people in the region, including many indigenous communities. The Pan-Borneo Highway and several hydroelectric dams are planned or already under construction in Sarawak, a Malaysian state comprising part of the Borneo. This development seeks to enhance economic growth and regional connectivity, support community access to services, and promote industrial development. However, the implications of the development of highway and dams for forest integrity, biodiversity and ecosystem services remained largely unreported. We assessed these development projects using fine-scale biophysical and environmental data and found several environmental and socioeconomic risks associated with the projects. The highway and hydroelectric dam projects will impact 32 protected areas including numerous key habitats of threatened species such as the proboscis monkey (*Nasalis larvatus*), Sarawak surili (*Presbytis chrysomelas*), Bornean orangutans (*Pongo pygmaeus*) and tufted ground squirrel (*Rheithrosciurus macrotis*). Under its slated development trajectory, the local and trans-national forest connectivity between Malaysian Borneo and Indonesian Borneo would also be substantially diminished. Nearly ~161 km of the Pan-Borneo Highway in Sarawak will traverse forested landscapes and ~55 km will traverse carbon-rich peatlands. The 13 hydroelectric dam projects will collectively impact ~1.7 million ha of forest in Sarawak. The consequences of planned highway and hydroelectric dams construction will increase the carbon footprint of development in the region. Moreover, many new road segments and hydroelectric dams would be built on steep slopes in high-rainfall zones and forested areas, increasing both construction and ongoing maintenance costs. The projects would also alter livelihood activities of downstream communities, risking their long-term sustainability. Overall, our findings identify major economic, social and environmental risks for several planned road segments in Sarawak—such as those between Telok Melano and Kuching; Sibu and Bintulu; and in the Lambir, Limbang and Lawas regions—and dam projects—such as Tutoh, Limbang, Lawas, Baram, Linau, Ulu Air and Baleh dams. Such projects need to be reviewed to ensure they reflect Borneo’s unique environmental and forest ecosystem values, the aspirations of local communities and long-term sustainability of the projects rather than being assessed solely on their short-term economic returns.

## Introduction

The forest of Borneo is one of the last remaining tropical forest strongholds on the planet. The island harbours 37 million hectares (Mha) of biodiversity-rich tropical forests [1] as well as extensive carbon-rich peatlands [2–4]. The majority of the 18 million inhabitants of Borneo rely on this forested landscape for their livelihoods, particularly indigenous peoples [5].

**Fig 1:**
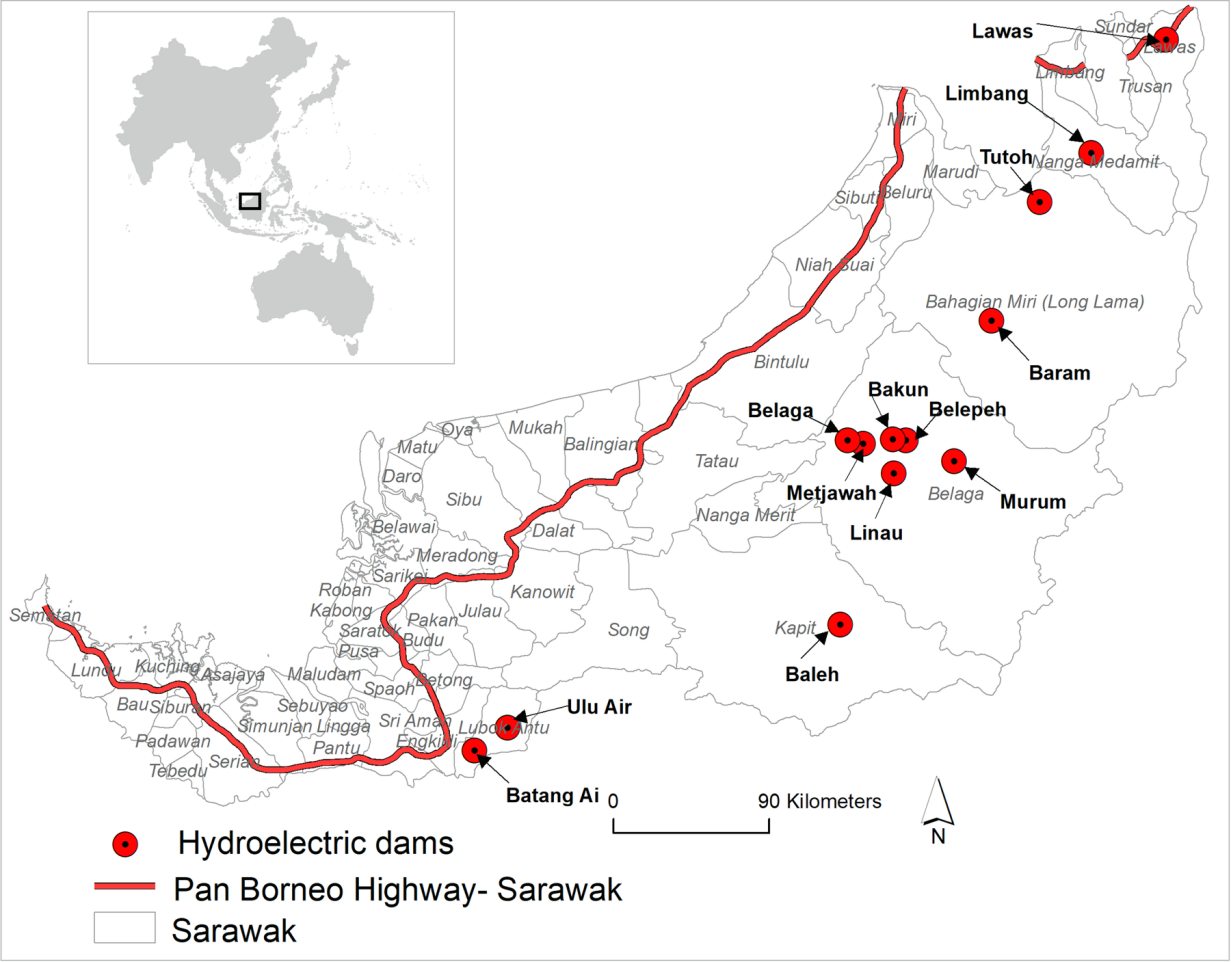
Major infrastructure expansion projects in Sarawak, Malaysia.

Borneo’s forest (Fig 1) is disappearing at a rate of 0.25 Mha per year [6]. It has lost more than 18 Mha of forest since 1973 [1]. Consequently, more than 600 vertebrate and plant species are threatened with extinction risk in the region [7]. The persistent loss of forest and biodiversity has been occurring due to agriculture expansion, industrial-scale logging, oil-palm plantations, illegal hunting, and the expansion of roads and other infrastructure, such as hydroelectric dams [1, 6, 8–10]. The state of Sarawak, in Malaysian Borneo, has lost eighty percent of its primary forest over the last forty years [1].

Sarawak continues to implement several infrastructure expansion projects, mainly roads and hydroelectric dams, to enhance economic growth (Fig 1). The Pan-Borneo Highway, for instance, spans Sarawak from southwest to northeast with a total length of 1,060 km, with an estimated finish date of 2021 [11, 12]. This highway project includes both the construction of new roads and upgrades to current roads, particularly the expansion of two-lane single carriageways to four-lane dual carriageways [11, 13]. Twelve planned hydroelectric dams occur in currently forest areas in southern and northeastern Sarawak and are scheduled to be completed by 2030 [14, 15] with one dam already operational in southwestern Sarawak [14].

The Sarawak dams are being constructed to supply electricity for planned industrial developments and to export electricity to nearby Indonesian Borneo and Sabah, Malaysia [14]. The Pan-Borneo Highway seeks to promote several societal benefits, including development of an industrial corridor, enhanced regional transportation connectivity, easier access to social services such as schools, markets, and hospitals, and increased access to natural resources such as timber and minerals [16]. It is expected that dam construction and assurances of electrical power supply to industries, and an improved regional connectivity via the highway, will facilitate large-scale economic investment in Sarawak. This investment will trigger several industrial developments and could have substantial benefits for the Sarawak economy [17].

The roads and dams in forested landscapes may, however, also generate numerous negative environmental, economic and social impacts [18–20]. These could be particularly severe in the context of Borneo, given its global conservation significance. Some of these impacts could entail irreversible losses of protected-area effectiveness, forest connectivity, critical habitat for biodiversity, and forest-carbon storage [21–25]. Such developments may also displace local communities and indigenous groups [14, 26] and burden the national economy with unanticipated debts [27, 28].

Ensuring habitat connectivity and minimal disturbance to forest areas in Sarawak is important for Borneo as a whole. Borneo hosts several iconic species—such as Bornean orangutans (*Pongo pygmaeus*), pygmy elephants (*Elephas maximus borneensis*), bearded pigs (*Sus barbatus*) and sun bears (*Helarctos malayanus*)—which require large home range to maintain viable populations [29, 30]. The forests in Sarawak facilitate habitat connectivity between Indonesian Borneo and other forests in Borneo, helping to sustain many iconic species in Borneo. Any impacts of the highway and dams on habitat connectivity and forest integrity in Sarawak are likely to have wider impacts on Borneo’s forests and wildlife populations. The impacts of these developments could also undermine trans-national conservation initiative in the region [31].

Although the anticipated benefits of the highway and hydroelectric dam projects in Sarawak have been widely highlighted [16, 17], the environmental, social and economic impacts from these projects have remained largely unreported for both Sarawak and Borneo broadly. It is therefore difficult to appraise the relative costs and benefits of these development projects. For instance, some studies (e.g. [14]) have warned that the planned dams in Sarawak do not fulfil sustainability criteria and that they lack in-depth information on the scale and nature of impacts on forested landscapes, ecosystem services and biodiversity. Here, we evaluate the extent of the impact of the planned roads and dams in Sarawak on protected areas, biodiversity, forest integrity, carbon storage and long-term regional sustainability. We make recommendations to improve the overall sustainability of the planned road and dam projects and reduce their environmental, social and economic impacts.

## Methods

### Roads and hydroelectric dams

We obtained spatial data on planned and ongoing road and dam construction projects in Sarawak from a variety of regional and national sources in an endeavour to include the majority of the projects in our analyses. We digitized the Pan-Borneo Highway, Sarawak (Fig 1) from the project’s map [11, 32] following the procedures listed in Alamgir et al. [21, 23] and Sloan et al. [26]. The project map was prepared using fine scale biophysical data for the project design and implementation [11, 32]. The digitization process involved geo-referencing the source map and tracing the route of the highway into a Geographic Information System (GIS). Subsequently, we retraced the digitized road layer in Google Earth with reference to the existing road alignments to ensure the maximum locational accuracy. The locational error of the final digitized highways is estimated to be < 200 m relative to their ultimate alignment on the ground.

The spatial data of hydroelectric dams in Sarawak were extracted from Sovacool and Bulan [14], and Tryse [33]. Altogether, there are 13 hydroelectric dams (Fig 1). Of these, one (Batang Ai) is currently operational, construction of two (Bakun and Murum) has been recently completed, and the remaining 10 dams are in various stages of planning and construction [14, 34]. The relative priority of construction of these 10 dams are still unclear but Baleh dam is in the beginning stages of construction and Baram, Limbang and Tutoh dams to follow [35].

### Landscape connectivity

Landscape connectivity depicts the links between forest patches across a landscape. Connectivity is important for wildlife communities to shift from one suitable habitat to another across the landscape. We assessed the impacts of the highway and hydroelectric dams on landscape connectivity in Sarawak conducting a morphological spatial pattern analysis (MSPA) using Graphical User Interface for the Description of Image Objects and their Shapes (Guidos Toolbox 2.6 version 4) [36, 37], following Alamgir et al. [21, 23] and Sloan et al. [22]. The MSPA analysis delineated forest areas in Sarawak into distinct forest-landscape elements including core forest (forest ≥ 500 m from the nearest forest edge), connectivity forest (forest corridors that connect different core forest patches or different segments of a core-forest patch) and edge forest (forest < 500 m from an edge). We then identified the highway routes and location of the hydroelectric dams amongst these elements of forest connectivity. Subsequently, we estimated areas of each distinct forest elements within a 1-km buffer of the highway, and a 25-km radius of the dams in a GIS interface.

In MSPA, we defined forest cover using the latest forest cover data for Borneo [38], which are based on 30-m Landsat land-cover classifications [38] describing three forest classes: primary forest, selectively-logged forest, and regrowth forest. Primary forest is defined as relatively pristine forest without significant sign of human disturbance apparent in the Landsat imagery. Selectively-logged forest is described as forest which has been disturbed via selective, commercial-scale logging since 1973 [38]. Regrowth forest is described as forest that was likely young regrowth in 1973 and roughly resembles old-growth forest currently in terms of canopy structure [38]. For MSPA analysis we classified primary forest, selectively-logged forest and regrowth forest as ‘forest class’ following Alamgir et al. [21]. Data were resampled to 100-m resolution for purposes of analysis. We used a 500-m threshold to define ‘edge effects’. This threshold is conservative, as edge effects can extend up to several kilometres into a forest interior [18]. Thresholds of 500 to 1000-m are widely used in road-impact research [21, 23, 39, 40].

Beyond landscape connectivity, we also assessed the likelihoods that the highway and hydroelectric dams may impact current forest cover in Sarawak. For this purpose, we classified forest into primary and selectively-logged forest and spatially overlaid the highway routes and hydroelectric dams’ location. Then we spatially assessed primary and selectively-logged forest areas within a 1-km buffer of the highway and a 25-km radius of the dams in a GIS interface. For this analysis, we used forest cover data from Gaveau, et al. [38] as described above and combined selectively-logged forest and regrowth forest into a ‘selectively-logged forest’ class.

### Protected area, peatland and slopes

We assessed the highway and hydroelectric dams with respect to protected areas, peatland and slopes. As such, we spatially overlaid the highway routes and hydroelectric dams’ locations on the above landscape features across Sarawak in a GIS interface. The protected area data were extracted from Global Forest Watch [41] and included both gazetted and ungazetted (proposed) protected areas in Sarawak. These were comprised of different forest designations, such as national parks, wildlife sanctuaries and forest reserves. These data are more robust at a local scale than other available protected area data as they are based on the protected area maps of the Sarawak Forestry Department [41]. The habitat extant data for Bornean orangutans, Sarawak surili (*Presbytis chrysomelas*) and proboscis monkey (*Nasalis larvatus*) were obtained from IUCN [7]. We obtained peatland data for Sarawak from the latest mapping of tropical peatland and wetland [42, 43], which is considered more updated and robust than other available peatland data (such as Page et al. [44]). We estimated slope across Sarawak from a widely used Global Multi-resolution Terrain Elevation Data 2010 (GMTED 2010) at 250-m spatial resolution [45]. We also obtained rainfall data for the wettest annual quarter for Sarawak at 1-km^2^ resolution [46]. We then evaluated the route profile of the highway with respect to slope and rainfall in a GIS interface.

## Results

### Infrastructure and protected areas

**Fig 2:**
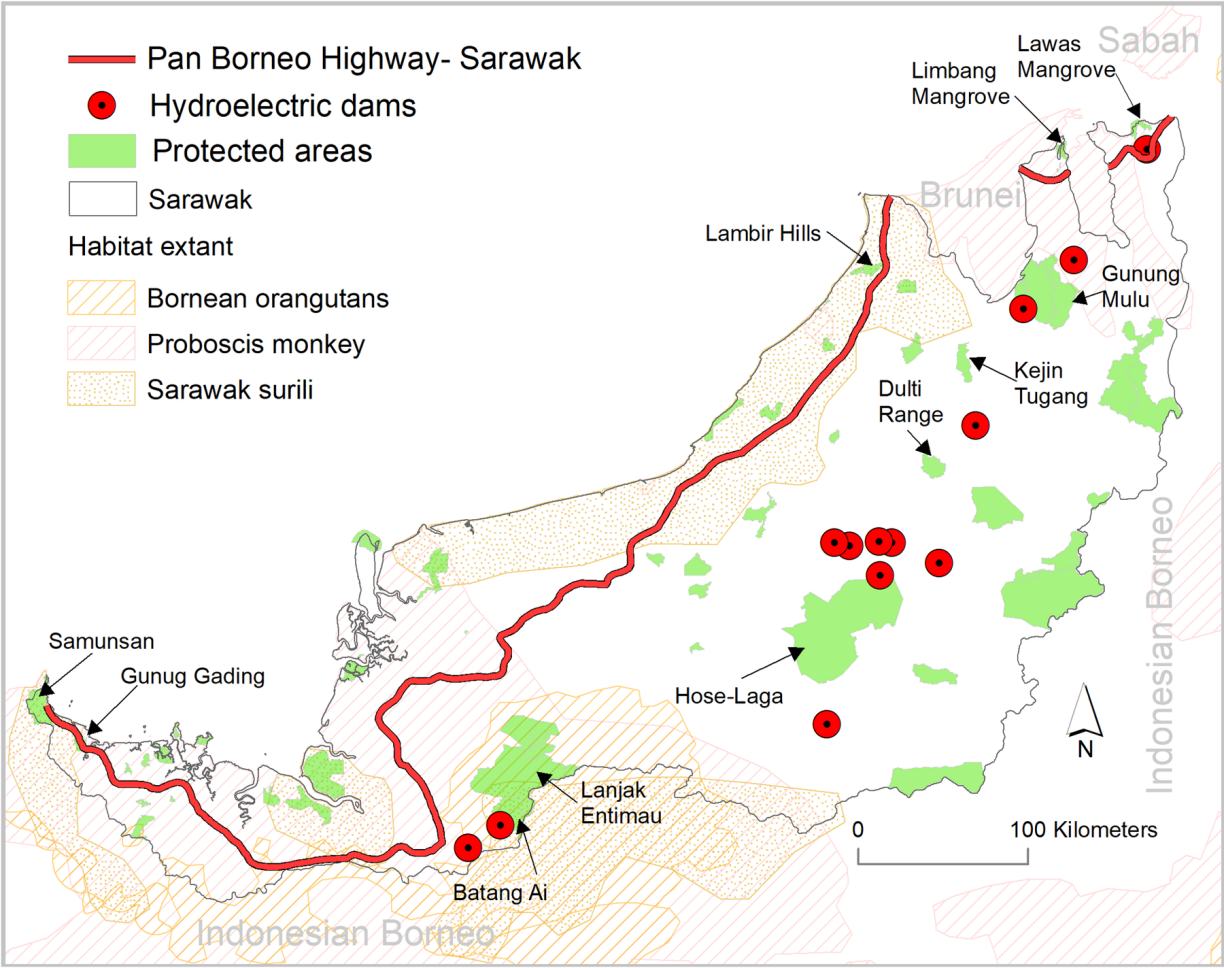
The Pan-Borneo Highway and hydroelectric dams in Sarawak penetrating into protected areas and key habitats of threatened species in Borneo.

Combined, the Pan-Borneo Highway and hydroelectric dams will affect 32 protected areas in Sarawak. The Highway will dissect three protected areas and 24 more protected areas will be within 25 km of the highway. Three additional protected areas are within the development periphery of the dams and four more protected areas are within the 25-km radius of the dams (Fig 2).

Notably, an ~11 km section of the highway will traverse the Samunsan Wildlife Sanctuary in western Sarawak—the oldest wildlife sanctuary in Sarawak and home to the endangered proboscis monkey and critically endangered Sarawak surili [7]. The Wildlife Sanctuary is also a critical habitat of tufted ground squirrel (*Rheithrosciurus macrotis*) which is only found in Borneo [47]. An ~8 km segment of the highway in the same region (Fig 2) will pass through the centre of the Gunung Gading National Park, a home to the world’s largest flowering plant species—the Rafflesia (*Rafflesia*) [48]. The Lanjak Entimau Wildlife Sanctuary—which contains most of the remaining habitat of the endangered Bornean orangutan in Sarawak—is within the 25 km buffer of the highway (Fig 2). A ~5 km section of the highway in northern Sarawak will divide the Lambir Hills National Park—one of the world’s most ecologically diverse areas and exceptionally rich biodiversity area [49]. The Limbang Mangrove National Park, a critical habitat for the endangered proboscis monkey in northeastern Sarawak is within the 25 km periphery of the highway (Fig 2).

Ulu Air, Linau and Tutoh hydroelectric dams will be located within the territorial boundary of the Batang Ai, Hosa-Laga and Gunung Mulu protected areas respectively (Fig 2). Lanjak Entimau Wildlife Sanctuary is within the 25 km periphery of Ulu Air while Hose-Laga National Park is within the 25 km radius of six hydroelectric dams—Baleh, Belaga, Metjawah, Bakun, Beleph and Murum. Dulti Range and Kejin Tugang National Park are within the 25 km radius of Baram hydroelectric dam while Gunung Mulu and Lawas Mangrove National Parks are within 25 km range of the Limbang and Lawas hydroelectric dams respectively (Figs 1 and 2).

### Infrastructure and forest connectivity

**Fig 3:**
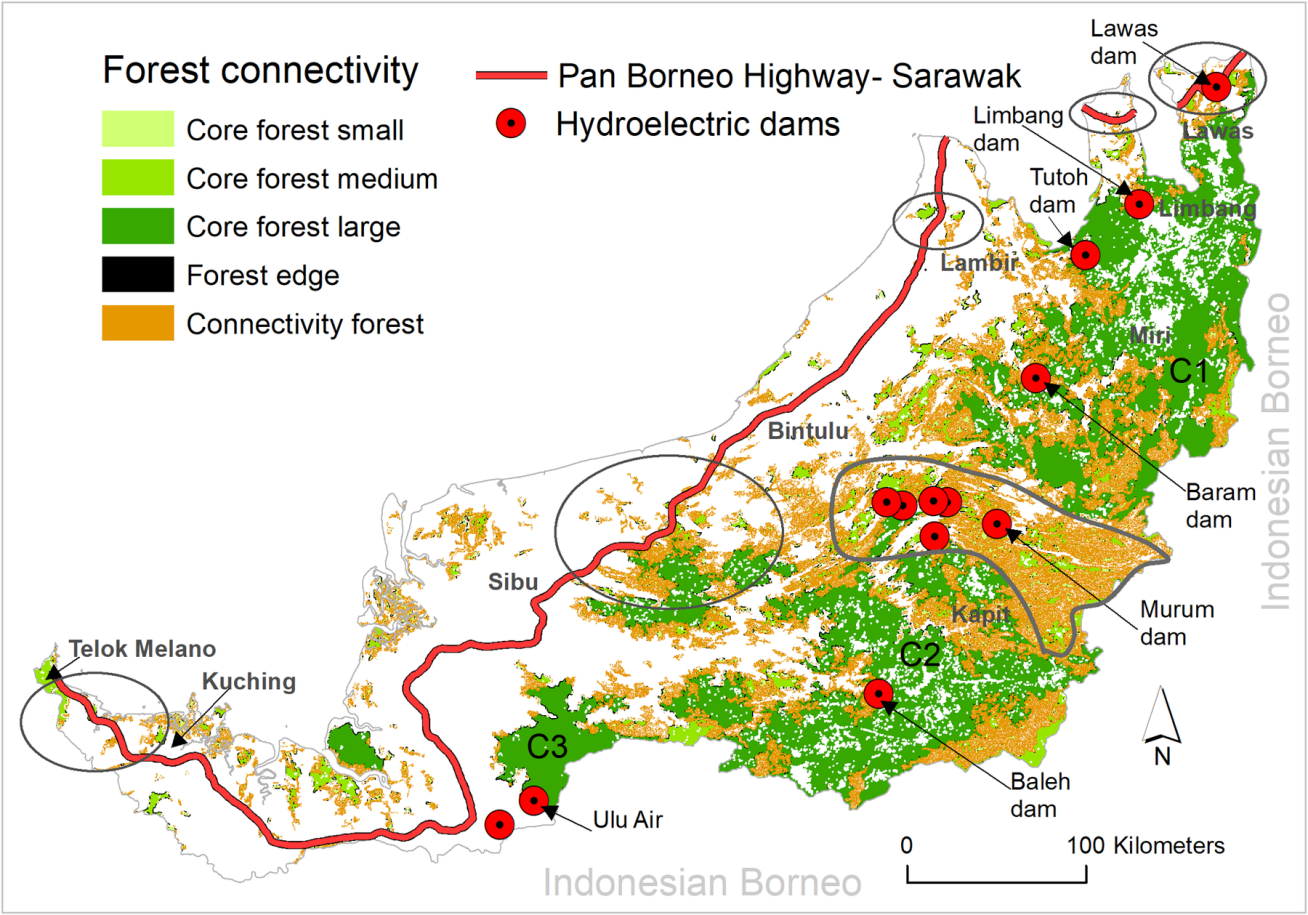
The Pan-Borneo Highway, hydroelectric dam projects and forest connectivity in Sarawak, Malaysia. C1, C2, and C3 indicates the three largest continuous core forest blocks. Circular highlights along the highway indicate the critical road segments threatening forest connectivity. Circular highlight in kapit region indicates the most important connectivity forest which will be impacted by the hydroelectric dams. Small, medium and large core forest indicates core forest block with an area of < 1,000 ha, 1,000 to 25,000 ha, and > 25,000 ha respectively.

The Pan-Borneo Highway and hydroelectric dams in Sarawak will reduce current forest connectivity and the area of core forest substantially. Using a conservative consideration that the highway will impact only on the 1-km spatial extent buffering the road, ~18,500 ha of connectivity forest and ~5,200 ha of core forest would be lost (Fig 3). Again assuming conservatively that the dams will impact only on the area within the 25 km radius of their development site, ~662,000 ha of connectivity forest and ~743,000 ha of core forest would be impacted (Fig 3).

Our analysis suggests that five segments of the highway will pose a critical threat to connectivity forest and core forest (Fig 3, circular highlights). These sections are a ~50 km road section between Telok Melano and Kuching, a ~110 km road section between Sibu and Bintulu, a ~30 km road section in Lambir, a 31 km road section in Limbang, and a ~40 km road section in Lawas region. Each segment would dissect connectivity forest and/or core forest in their respective regions of Sarawak.

A number of dams also critically threaten connectivity and core forest areas. These include the three largest remaining blocks of core forest (C1, C2 and C3 in Fig 3), which will be impacted by several dams. The Baram, Tutoh, and Limbang dams will be located in the largest remaining core forest block (C1), while the Baleh and Ulu Air dams will be located in the second largest (C2) and third largest (C3) remaining blocks of core forest, respectively (Fig 3). Among the thirteen dams, Baleh dam will impact the most core forest area (~109,000 ha) followed by Tutoh (~97,000 ha), Limbang (92,000 ha) and Baram dam (74,000 ha). Six dams—Belaga, Metjawah, Bakun, Belepeh, Linau and Murum—will be located in an area containing several stepping stones of core forest and the largest remaining connectivity forest block in Sarawak (Fig 3). The Murum dam will impact the most connectivity forest (~107,000 ha), followed by Belepeh (~96,000 ha) and Bakun dam (~81,000 ha) (Fig 3). The connectivity forest in the Kapit region that will be impacted by six dams is the only remaining forest connectivity found between the core forest block in northeastern Sarawak and southwestern Sarawak. Beyond that, this connectivity forest also supports transnational forest connectivity between the Sarawak component of Malaysian Borneo and Indonesian Borneo (Fig 3).

### Infrastructure and peatland

**Fig 4:**
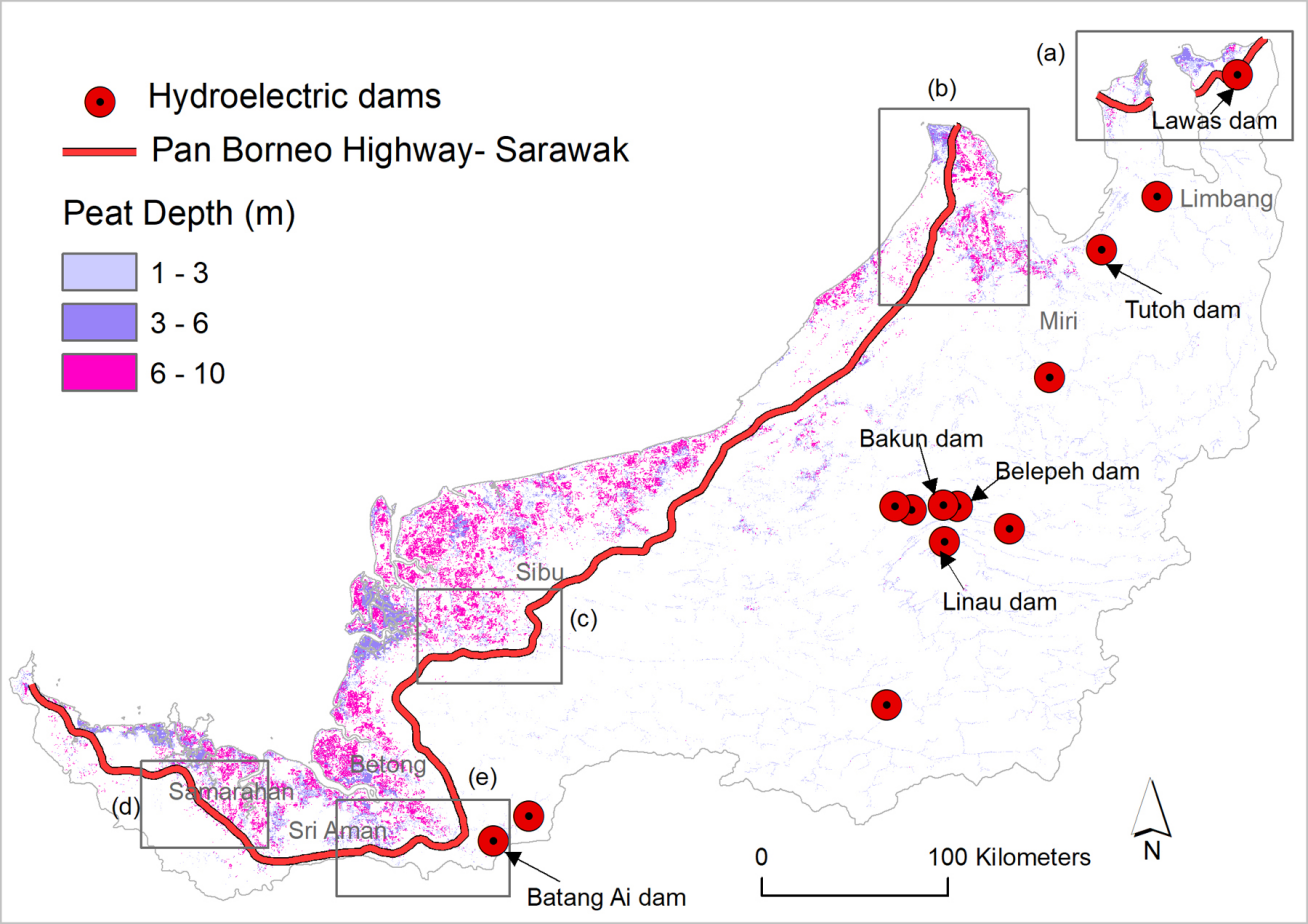
The Pan-Borneo Highway and hydroelectric dam projects with respect to peatlands in Sarawak, Malaysia.

The Pan-Borneo Highway and hydroelectric dams in Sarawak are likely to substantially impact peatland across the region. Conservatively considering that the highway will impact only a 1-km buffer on both sides of the road, we found that ~11,000 ha of peatlands will be degraded, with ~65% being deep or very deep (> 3m) and thus having large carbon stocks (Fig 4). Assuming that the dams will only impact on peatlands that are located within a 25 km radius, we estimate that ~80,000 ha of peatlands will be impacted, including more than 10,000 ha of deep and very deep peatland (Fig 4).

More than 55 km of the Pan-Borneo Highway in Sarawak will traverse peatland, including ~39 km in deep and very deep peatland (> 3m) areas. A number of road segments will impact peatland habitats—such as a ~30 km and a ~50 km road section in Limbang (Fig 4, marked ‘a’), a ~ 66 km road section in Miri (Fig 4, marked ‘b’), a ~90 km road section in Sibu (Fig 4, marked ‘c’), a ~80 km road section in Samarahan (Fig 4, marked ‘d’) and a ~80 km road section in Sri Aman region (Fig 4, marked ‘e’).

The extent of the impacts on peatland will be different for each dam. Out of the 13 dams, Lawas dam will have the greatest impact on peatland, followed by Linau, Bakun, Beleph and Tutoh dam, with each dam respectively impacting ~10,000 ha, ~9,200 ha, ~8,600 ha, ~8,200 ha and ~7400 ha of peatland habitat. Notably, Tutoh dam will impact a very large block of deep/very deep peatland in northern Sarawak (Fig 4).

### Infrastructure and forested landscapes

**Fig 5:**
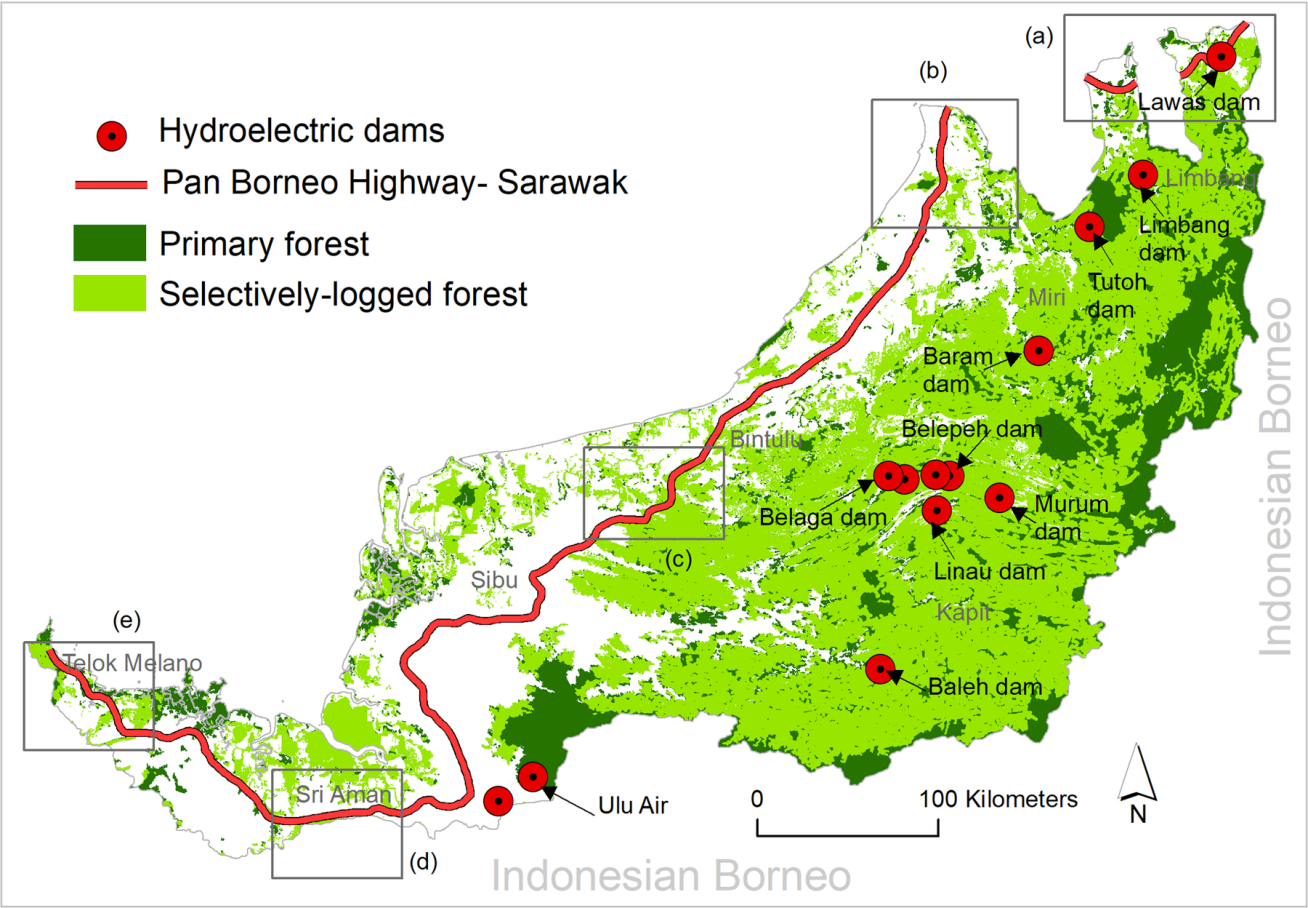
Pan-Borneo Highway and hydroelectric dam projects with respect to current forest distribution in Sarawak, Malaysia.

The Pan-Borneo Highway and developing hydroelectric dams in Sarawak will impact forested landscape extensively. The highway will, for instance, impact ~ 9,000 ha of forest which includes ~6,500 ha of primary forest, conservatively assuming the highway impacts only the area within the 1-km buffer on each side. The dams will impact ~1.7 million ha of forest including ~350,000 ha of primary forest, also assuming the dams’ impacts occurring only within a 25 km radius of their development site (Fig 5). Notably, if all dam projects proceed as planned, the impacts of these dams on forests in regards to forest loss will be much higher than the highway. However, the highway construction is happening at a faster rate than dam construction and limited information is available in the literature regarding the priority of the construction of the planned dams but aims to finish by 2030.

In total ~161 km of the highway will traverse forested landscapes of which ~125 km will travel through primary forest and ~36 km through selectively-logged forest. Specifically, two road segments in the Limbang region (Fig 5, marked ‘a’), a road segment in Miri region (Fig 5, marked ‘b’), a road segment between Sibu and Bintulu (Fig 5, marked ‘c’), a road segment in Sri Aman (Fig 5, marked ‘d’) and a road segment in Telok Melano (Fig 5, marked ‘e’) will extensively dissect current primary and selectively-logged forest (Fig 5).

A number of dams will impact heavily upon currently forested landscapes. Baleh dam, for instance, will impact the highest amount of forest area (~180,000 ha) followed by Baram (~170,000 ha), Limbang (~157,000 ha), Linau (~155,000) and Tutoh dam (~151,000 ha). Ulu Air dam will impact the highest amount of area currently containing primary forest (~64,000 ha) followed by Tutoh (~61,000 ha) and Limbang dam (~44,000 ha) (Fig 5).

### Infrastructure and landslides

**Fig 6:**
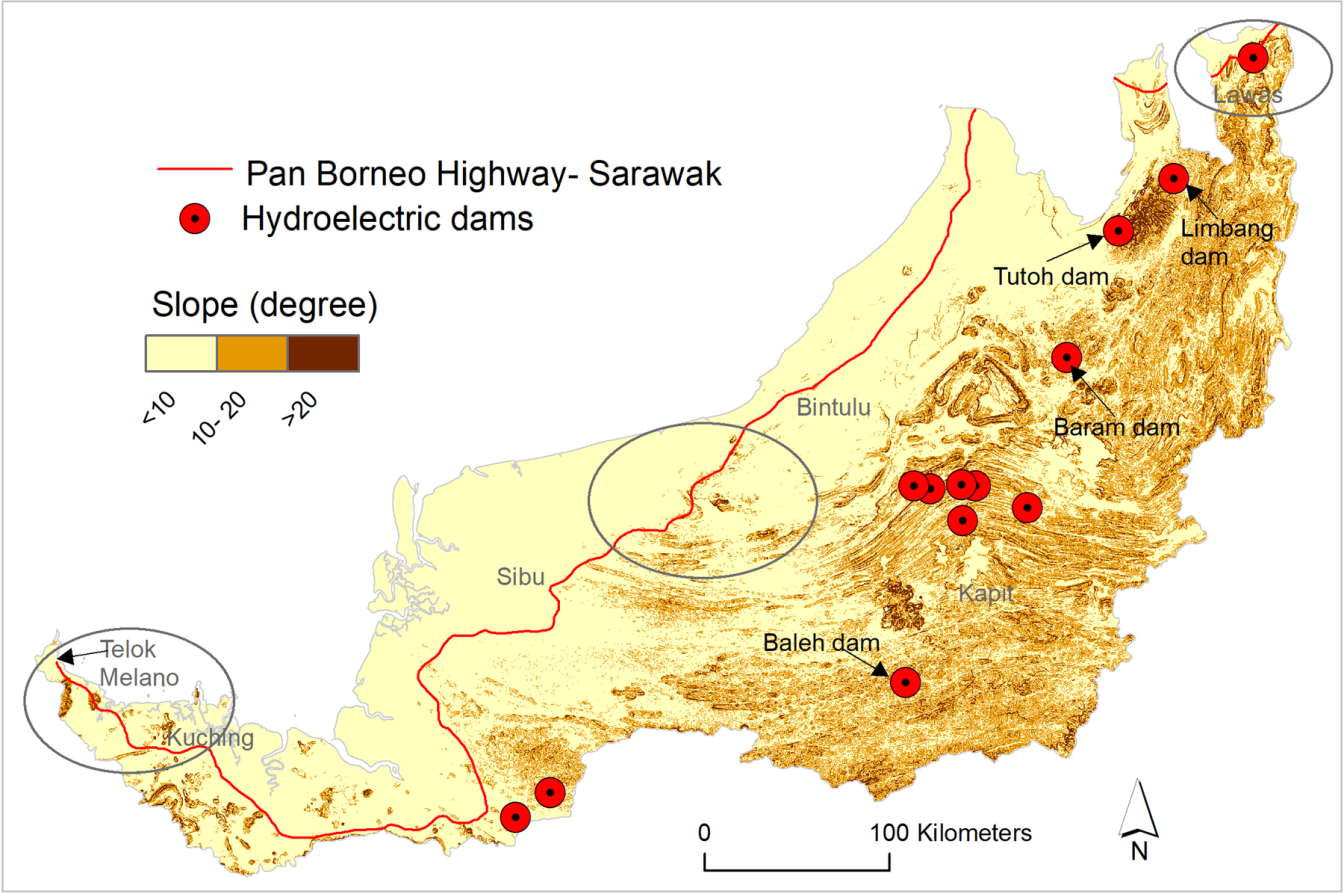
Pan-Borneo Highway and hydroelectric dams in Sarawak and imminent frontiers of landslides. The highway and hydroelectric dams with highly vulnerable landslide locations (as circled).

**Fig 7:**
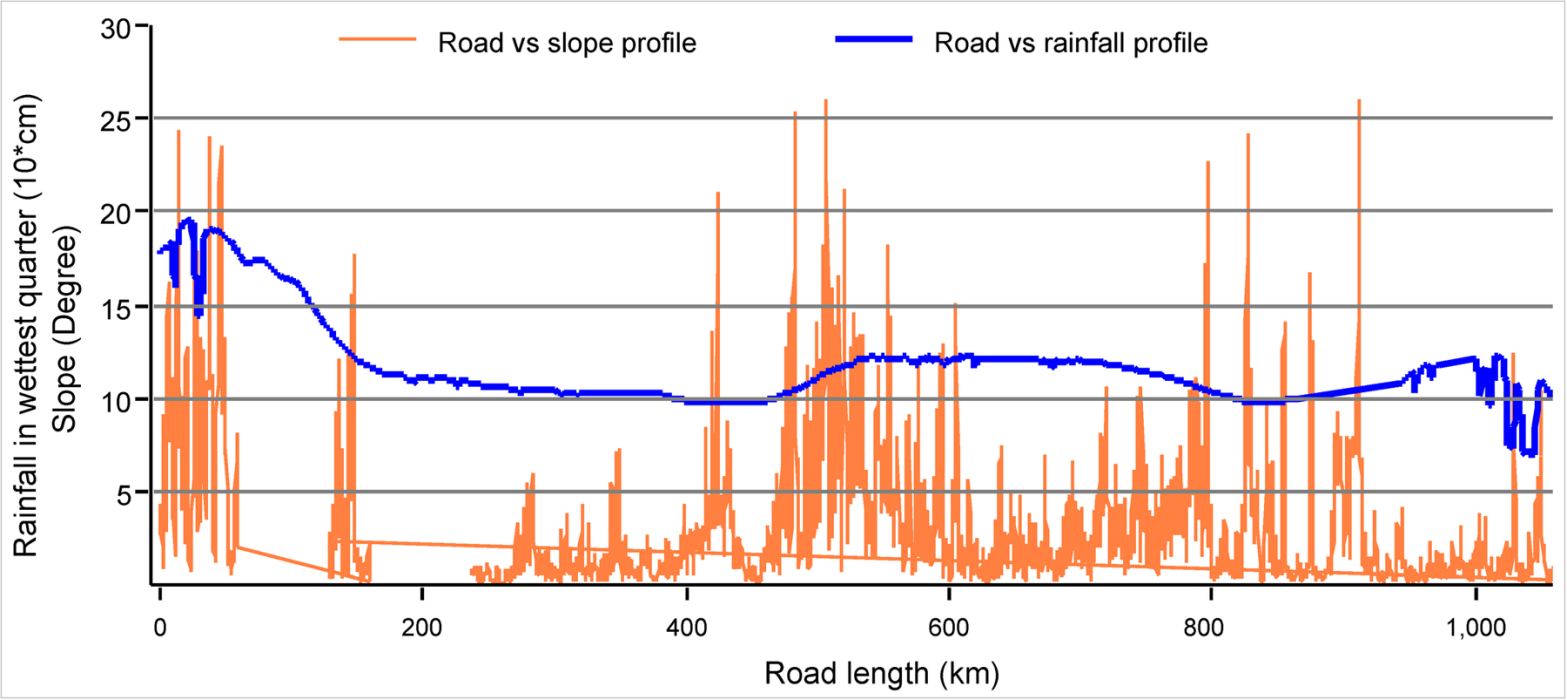
Pan-Borneo Highway Profile with respect to slope and total rainfall in wettest quarter.

The current route of the Pan-Borneo Highway will likely result in increased landslides in Sarawak. By mapping the highway against slope and total rainfall in the wettest quarter (Figs 6 and 7), our results suggest that several road segments will traverse steep slopes as high as > 20 degrees and in areas where the wettest quarter of rainfall is as high as > 1500 mm (Figs 6 and 7)—a zone highly vulnerable to landslides. Most of these areas are currently forested (Figs 5 and 6) and would thus require deforestation and site disturbances for road construction, making these sites more vulnerable to landslides, both during initial development stage and post development. If the roads are built as planned, it is likely that high rainfall (Fig 7) will trigger frequent and often large-scale landslides in the region. In this regard, a road segment between Sibu and Bintulu appears the most risky followed by a road segment between Telok Melano and Kuching, and a road segment in Lawas region (Fig 6, circular symbols). Frequent and large-scale landslides in the aftermath of high rainfall along the highway in Sibu, Bintulu and Limbang region have already been reported [50, 51].

Many planned and ongoing hydroelectric dams in Sarawak will also increase landslides in the region. If dam construction proceeds as planned, all dams will be constructed in currently forested areas and will inundate forest growing on steep slopes (Figs 5 and 6). Water-level rise and resulting in increased geological pressure in the area, forest inundation, disturbances and deforestation for the dams and associated infrastructure would all likely create favourable conditions for frequent and large-scale landslides, as observed to have occurred elsewhere in the region [52]. Specifically, the Tutoh dam and Limbang dam would be particularly dangerous as those dams are set to be constructed in a region of very steep slopes (> 20 degree) and which are currently covered by relatively intact forest (Figs 6 and 5).

The above mentioned implications will greatly increase the maintenance cost of the highway and relevant disaster-management costs in the region in the post-hydroelectric dams’ development. The relevant organization such as Sarawak Government should consider to trigger a full cost-benefit analysis of the projects considering the maintenance cost of the roads and dam projects and associated disaster-management costs.

## Discussion

### Borneo’s biodiversity

Borneo is one of the richest biodiversity strongholds on the planet. The Pan-Borneo Highway and hydroelectric dams in Sarawak substantially threaten the remaining biodiversity of the region through several means—direct habitat loss through forest clearing, forest inundation, dissecting protected areas and key habitats, habitat fragmentation, core-forest degradation, and habitat isolation (Figs 2, 3 and 5). Negative impacts of infrastructure expansion on biodiversity have already been reported from several areas in the region, such as nearby Sabah, Malaysia[31], Indonesian Borneo [21] and New Guinea [23, 26].

Many wildlife species will become more vulnerable to population loss under the planned projects, greatly increasing local extinction risk, including of several globally endangered species: proboscis monkey, Bornean orangutans and Sarawak surili. The highway would threaten the remaining protected key habitats of proboscis monkey in Sarawak—Samunsan, Limbang and Lawas protected areas—and a key protected habitat of the Sarawak surili, Samunsan protected area (Fig 2). In fact, the entire last remaining habitat—Lanjak Entimau protected area—of the endangered Bornean orangutans in Sarawak would be imperilled by the highway and Ulu Air hydroelectric dam.

Apart from such habitat loss and degradation as mentioned above, the wildlife population in the region would also suffer from the increased access to the region the infrastructure would provide to subsistence hunters and poachers and, post-construction, an increased chance of wildlife vehicle collisions. A drastic decline of the population of many wildlife species in the region caused by hunting is evident [53, 54]. The infrastructure expansion projects would further exacerbate those losses. This presupposition is based on past evidence from Sumatra orangutans (*Pongo abelii*) and African forest elephants (*Loxodonta cyclotis*) whose populations declined substantially due in part to road incursions in their habitat and resultant their habitat clearance mostly by illegal logging and deforestation [55, 56]. Habitat inundation due to hydroelectric dams may also risk wide spread mammal extinctions in the region, as has occurred in parts of the lowland Amazonia forest area due to a hydroelectric dam project [24]. It is estimated that only three dams—Bakun, Murum and Baram—in the region would inundate habitat of three million birds, 110 million mammals, and 900 million trees [57].

### Forest integrity and protected area effectiveness

The extent of Bornean forest has been declining at an alarming rate since industrial development on the island [9]. The identified infrastructure development projects will further accelerate forest loss in the region (Figs 3 and 5). For instance, several segments of the highway will traverse primary forest or selectively-logged forest, and a number of hydroelectric dams will be located in primary forest and or selectively-logged forest potentially inundating a vast area of forest in the region (Figs 3 and 5). The highway would cause forest loss directly via deforestation during the construction stage and through industrial development thereafter. The dams would cause forest loss and degradation primarily by the inundation of forest, leading to large-scale forest mortality and additional secondary conversion associated with infrastructure expansion. Several million hectares of forest in Borneo have been lost over recent decades, Sarawak alone has lost > 2 million ha since the early 1970s while an additional > 5.2 million ha have been logged [9]. Sarawak currently retains only 7 million ha of forests with the majority of this extent formed by selectively-logged forests (5.2 million ha) though a few blocks of primary forests do remain (1.8 million ha) [9] (Fig 5). Each of the proposed dams would likely impact more than 100,000 ha of forest. For instance, the Baleh dam is estimated to likely impact 180,000 ha of forest while the Tutoh dam would impact more than 150,000 ha of forest including 60,000 ha of primary forest. This plausible scenario is already apparent in Sarawak as the completed Bakun and Murum dams have inundated thousands of hectares of forest in the region [14].

Lowland forest in Sarawak has experienced extensive clearing due to previous developments. Therefore, most of the primary forest and selectively-logged forests in Sarawak are restricted to the upland areas [9]. These upland forests would be vulnerable to future deforestation and forest conversion under the planned hydroelectric dams and their associated infrastructure and industrial developments. Similarly, much of the remnant lowland forests of Sarawak would potentially be lost due to the highway and associated development in the near future.

Malaysia is working to expand its protected-area network and has stressed in its official planning documents that an effective protected-area network is the backbone of sustainable biodiversity and ecosystem service management in Borneo [58]. Our results suggest that planned and ongoing infrastructure expansion projects will significantly diminish the effectiveness of several protected areas in terms of biodiversity conservation and ecosystem service management. This diminishment will occur as several road sections will be within and in the periphery of protected areas, and several dams will inundate a number of protected areas including Gunung Mulu, a UNESCO World Heritage site (Fig 2). Roads in protected areas and in their periphery are particularly concerning as, even if forests are retained, they are well known to dramatically reduce habitat quality [59].

In addition to several key protected areas for endangered species (mentioned in the above biodiversity section), some protected areas that would be impacted from these infrastructure projects such as Batang Ai and Lanjak Entimau extend into Indonesian Borneo (Fig 2). As such, impacts on these protected areas would have a considerable transboundary effects. These impacts would violate the Malaysia National Policy on Biological Diversity, which highlights the significance of transboundary protected areas [58]. Furthermore, Malaysia intends to increase terrestrial protected areas, targeting a coverage of 20 percent of its land area by 2025 [58], potentially including several blocks of remaining primary forest. A vast majority of the remaining primary forest would however, be impacted by the highway and dams (Fig 5). As such, those forest blocks, which may currently have the potential for protected area listing, may become degraded and not be able to provide suitable habitat for the biodiversity if declared protected in the future. Consequently, the ongoing and planned infrastructure projects would undermine Malaysia’s conservation efforts—to both increase the effectiveness of the current protected areas and also to increase effective protected areas in future.

### Climate change

The current infrastructure expansion projects in Sarawak do not appear to factor in climate change threats as they would increase both the carbon footprint and ecosystem vulnerability to climate change of the region (Figs 4 and 5). The projects would have three major implications on the region’s carbon footprint. First, the highway segments, dams, associated infrastructure and future development in forested areas would require large-scale deforestation leading to extensive greenhouse gas emission to the atmosphere. For instance, Bakun dam would require the clearing of 50 million m^3^ of forest biomass [14].

Second, several sections of the Pan-Borneo Highway will traverse large tracts of peatland (Fig 4). Road construction across this peatland would emit considerable amounts of greenhouse gases to the atmosphere as these peatlands often have extremely high carbon stocks, much more than most other terrestrial ecosystems [60]. The remaining peatland in the periphery of the highway would be more vulnerable to future disturbances, such as fire, as has previously occurred in nearby Indonesian Borneo [61]. The environmental consequences of peat burning in Indonesian Borneo was felt extensively both within the country and across the Southeast Asian region [61].

Third, as each dam would inundate thousands of hectares of forest (Fig 5). These forests will eventually decay in the warm tropical temperatures under both aerobic and anaerobic conditions [20]. Most of the lost forest biomass will be converted into carbon dioxide and methane [14].

The planned and ongoing infrastructure expansion projects would increase the vulnerability of plant and animal species to climate change by reducing forest connectivity, core forest habitat and habitat degradation (Figs 2, 3 and 5). Several plant and animal species are predicted to move latitudinally or elevationally in response to climate change [62–65]. Moreover, several wildlife species in Borneo require large home ranges to maintain viable populations, such as Bornean orangutans, bearded pigs and sun bears [29, 30]. Much of this range spans both Indonesian and Malaysian Borneo. Consequently, maintaining appropriate forest connectivity and large block of core forest habitat in the landscape is essential to facilitate both plant and wildlife movement under future climate change scenarios. However, the current infrastructure expansion projects would destroy or severely degrade both connectivity forest and core forest areas substantially in the region.

The highway would also reduce connectivity forest that currently connects several small patches of core forest areas vital for climate change adaptation measures for a number of globally endangered species in Borneo such as proboscis monkey, Sarawak surili and Bornean orangutan (Fig 3). Apart from inundating (at least partially) the three large blocks of core-forest areas in the region (Fig 3)—critical for providing habitat for plants and animals—the dams would also inundate several stepping stones of core forest areas in Kapit region that are vital for landscape connectivity in light of climate change adaptation measures. This would potentially isolate currently connected southwestern Sarawak, northeastern Sarawak and Indonesian Bornean forests. This impact would be particularly threatening, as a substantial decline in connectivity forest, core forest and stepping-stone habitat from the development of hydroelectric dams has already been reported from similar forests in the region and elsewhere [14, 19].

### Risks of long-term unsustainability

Several sections of the highway and a number of dams would potentially foment long-term unsustainability in Sarawak and beyond. In addition to the likely cost to biodiversity, forest cover, and climate change mitigation, as discussed above, several ongoing economic and social challenges are also plausible in the context of infrastructure development.

Road construction costs in tropical areas like Borneo are usually high due to environmental factors [18]. Moreover, construction of the roads over peatland (Fig 4), and dams, roads and associated infrastructure on extremely steep slopes (Figs 6 and 7) will further increase the costs of these developments. The dam construction projects in Sarawak are amongst the most capital-intensive projects in South East Asia, yet their funding is still unclear [14]. The planned funding sources include debt markets, multilateral donors, private investment and state and federal government [35]. Malaysia is already endeavouring to borrow heavily from debt markets to finance the Pan-Borneo Highway [66]. Borrowing heavily from debt markets for the highway and dam projects will raise Malaysia’s debt burden to an unprecedented level [66]. Large loans from multilateral lenders were tainted with project unsustainability and uncertainty, and Malaysia consequently halted or cancelled several multilateral donor-funded infrastructure projects due to poor governance, debt, and environmental concerns [67, 68]. Cumulatively, some scientists have warned that the construction of mega-hydropower dams are too costly to build in a sustainable way that can deliver net positive economic outcomes [69]. Beyond high initial costs, in the aftermath of construction, frequent landslides (Figs 6 and 7) and higher maintenance costs of these infrastructure projects may impose additional burdens on the national economy through ongoing expenditure requirements.

The highway segments and dams in upstream forested areas (Figs 6 and 7) may negatively impact the livelihood activities of downstream populations across Borneo. Millions of farmers, fishers, and indigenous communities across Borneo have livelihoods directly depend on forest and river integrity in the region. Local communities have already voiced concerned regarding the impacts of these planned developments on their livelihood activities [34].The highway segments and dams in upstream forested areas, subsequent forest clearance and disturbances, and associated infrastructure development will deteriorate water quality with increase sediment loads in Borneo’s river systems, in addition to flooding vast forest and farm lands. A consequence widely reported in similar tropical areas [70] and evident in the region in the aftermath of construction of Bakun and Murum dams [14, 34]. Additionally, the impacts on livelihoods may entail economic opportunity costs in the millions of dollars for a protracted period of time [71].

Some of the infrastructure expansion projects may cause long-lasting social stress, particularly in indigenous communities. It can happen in various forms such as involuntary displacement from ancestral lands and generational poverty, cultural changes, limited availability of long-practiced livelihood activities, and frayed social relationships [18, 72]. While the highway will provide modest benefits—access to better road network—to the indigenous people, none of the dams would provide electricity to local people [14]. In fact, the dams would require a large-scale relocation of the local population, particularly the indigenous population. The Bakun and Murum dams required the relocation of 10,000 and 3,400 indigenous people respectively, the majority of which was done involuntarily [14]. The wellbeing of previously-relocated indigenous populations has declined since the resettlement because of the lack of internalizing indigenous population’s aspiration in the project structure and resultant failure of the projects and associated policy commitments to provide required financial and social supports to the indigenous populations [72]. Accordingly, indigenous communities are largely oppositional towards several proposed hydroelectric dams in Sarawak, as evident by various protests [73, 74]. In fact, local protests led by indigenous communities have resulted in a moratorium on the Baram dam, following the construction of a related access road [75]. Furthermore, the involuntary displacement of indigenous communities also contradicts the U.N. Sustainable Development Goals (SDGs) [76], which Malaysia aspires to embrace in their developments [77].

### Conclusions and policy implications

Borneo forests possess both high conservation and natural-resource values. So, development projects in Borneo need to be less destructive for the environment, economically feasible—with low construction and maintenance costs—and socially acceptable—supporting the aspirations of local communities. Our results suggest that the current infrastructure expansion projects in Sarawak have overlooked the high conservation value of Borneo forests, climate change implications in the region, social aspirations, and the projects’ overall economic feasibility and long-term sustainability. Therefore, we suggest the following based on our analyses:

- The highway segments that will traverse protected areas, key habitat of threatened species and peatlands should be cancelled. These specifically include the road segments in the periphery of Samunsan, Gunung Gading, Lambir Hills, Limbang mangrove and Lawas mangrove protected areas, and the road sections in the periphery of the peatland in Miri, Sibu, Sri Aman and Samarahan regions. If the construction of these road segments is necessary for the development connectivity in the region then re-routing of the current routes through already cleared non-peatland areas is highly advisable.
- The road segments set to traverse unprotected primary or selectively-logged forest, connectivity forest and core forest areas should be considered as environmentally highly-risky. These include road segments between Telok Melano and Kuching, Sibu and Bintulu, Sri Aman, Miri and Lambir regions. These road segments should only proceed under tightly enforced and appropriate impact mitigation strategies such as declaring relevant zones as protected areas prior to road construction and increased environmental and social law enforcement thereafter.
- The road segments set to traverse forest areas with steep slopes (>20 degree) and high rainfall (>1500 mm wettest quarter rainfall) should be cancelled, greatly minimized or reconsidered in light of landslide hazards. These areas include road sections between Telok Melano and Kuching, Sibu and Bintulu and Lambing regions. Such road sections would entail high construction and maintenance cost and jeopardise livelihood, water quality, and ecosystem services for downstream populations.
- Very recently, the government plans to re-route the Pan-Borneo highway section in northern Sarawak so as to now go around Brunei thereby connecting Miri, Marudi, Limbang and Lawas. If this re-routing proceeds as currently initiated, the re-routed section would likely have an even bigger environmental impact in the region than as analysed and reported in the current study as the section would cut through the largest remaining and connected forest in northern Sarawak. As such, the re-routing of this section should not be considered as a viable option due to environmental concerns.
- The master plan of hydroelectric dam construction in Sarawak needs to be reviewed in accordance with the extensive potential impacts that could occur from each dam. Clearly, the current plans for the hydroelectric dams construction in Sarawak have considered neither the unique environmental values of the landscape nor long-term overall sustainability of the projects. Under these values, several hydroelectric dam plans appear unviable and should be cancelled including Tutoh, Limbang, Lawas, Baram, Linau, Ulu Air and Baleh dams.
- Finally, a full cost-benefit appraisal of the development projects should be undertaken by the relevant authority, such as Sarawak State Government. Such an appraisal should include not only construction costs and environmental costs but also the long-term costs of maintenance for the highway and hydroelectric dams, relevant disaster management (such as large-scale landslides), and support services for the wellbeing of relocated communities including indigenous communities.
